# Caveolin-1 accelerates hypoxia-induced endothelial dysfunction in high-altitude cerebral edema

**DOI:** 10.1186/s12964-022-00976-3

**Published:** 2022-10-17

**Authors:** Yan Xue, Xueting Wang, Baolan Wan, Dongzhi Wang, Meiqi Li, Kang Cheng, Qianqian Luo, Dan Wang, Yapeng Lu, Li Zhu

**Affiliations:** 1 Institute of Special Environmental Medicine, Co-Innovation Center of Neuroregeneration, Nantong University, Nantong, 226019 China; 2 Medical School of Nantong University, Nantong, 226007 China; 3 Nantong Health College of Jiangsu Province, Nantong, 226010 China; 4 Department of Hepatobiliary and Pancreatic Surgery, Affiliated Hospital of Nantong University, Nantong, 226006 China

**Keywords:** High-altitude cerebral edema, Caveolin-1, Blood‒brain barrier, Endothelium, Claudin-5

## Abstract

**Background:**

High-altitude cerebral edema (HACE) is a serious and potentially fatal brain injury that is caused by acute hypobaric hypoxia (HH) exposure. Vasogenic edema is the main pathological factor of this condition. Hypoxia-induced disruptions of tight junctions in the endothelium trigger blood‒brain barrier (BBB) damage and induce vasogenic edema. Nuclear respiratory factor 1 (NRF1) acts as a major regulator of hypoxia-induced endothelial cell injury, and caveolin-1 (CAV-1) is upregulated as its downstream gene in hypoxic endothelial cells. This study aimed to investigate whether CAV-1 is involved in HACE progression and the underlying mechanism.

**Methods:**

C57BL/6 mice were exposed to HH (7600 m above sea level) for 24 h, and BBB injury was assessed by brain water content, Evans blue staining and FITC-dextran leakage. Immunofluorescence, transmission electron microscope, transendothelial electrical resistance (TEER), transcytosis assays, and western blotting were performed to confirm the role and underlying mechanism of CAV-1 in the disruption of tight junctions and BBB permeability. Mice or bEnd.3 cells were pretreated with MβCD, a specific blocker of CAV-1, and the effect of CAV-1 on claudin-5 internalization under hypoxic conditions was detected by immunofluorescence, western blotting, and TEER. The expression of NRF1 was knocked down, and the regulation of CAV-1 by NRF1 under hypoxic conditions was examined by qPCR, western blotting, and immunofluorescence.

**Results:**

The BBB was severely damaged and was accompanied by a significant loss of vascular tight junction proteins in HACE mice. CAV-1 was significantly upregulated in endothelial cells, and claudin-5 explicitly colocalized with CAV-1. During the in vitro experiments, hypoxia increased cell permeability, CAV-1 expression, and claudin-5 internalization and downregulated tight junction proteins. Simultaneously, hypoxia induced the upregulation of CAV-1 by activating NRF1. Blocking CAV-1-mediated intracellular transport improved the integrity of TJs in hypoxic endothelial cells and effectively inhibited the increase in BBB permeability and brain water content in HH animals.

**Conclusions:**

Hypoxia upregulated CAV-1 transcription via the activation of NRF1 in endothelial cells, thus inducing the internalization and autophagic degradation of claudin-5. These effects lead to the destruction of the BBB and trigger HACE. Therefore, CAV-1 may be a potential therapeutic target for HACE.

**Video abstract**

**Supplementary Information:**

The online version contains supplementary material available at 10.1186/s12964-022-00976-3.

## Background

High-altitude cerebral edema (HACE) is a severe and potentially fatal manifestation of high-altitude illness, with an incidence of approximately 0.5–2% [1, 2]. HACE is considered a malignant transformation of acute mountain sickness (AMS) [3], but the mechanism of transformation is unclear. Limited by the lack of understanding of the pathogenesis of HACE, there is a paucity of clinically effective means to prevent HACE. Based on previous studies, the onset of HACE has been reported to be associated with altitude, the rate of altitude ascent, the duration of exposure to hypobaric hypoxia (HH), infection, mood, and genetic factors [4–7]. A previous study showed positive implications for rapidly reducing the altitude and adjuvant drug therapy in treating HACE [8]. However, due to the acute onset and rapid progression of HACE, patients often suffer severe sequelae if untreated in time. Therefore, it is important to understand HACE pathogenesis and develop practical and effective prevention measures.

Magnetic resonance imaging (MRI) data from HACE patients showed marked brain edema with microvascular haemorrhages in the corpus callosum and white matter areas, accompanied by cytotoxic and vasogenic edema [9, 10]. Clinical and animal studies have demonstrated that vasogenic edema induced by cerebral microvascular rupture due to blood‒brain barrier (BBB) injury is considered the leading cause of HACE [11–13]. The BBB is a highly selective semipermeable barrier existing between the brain and blood that plays a significant role in maintaining the homeostasis of the cerebral microenvironment [14]. It has been indicated that hypoxia can induce BBB injury in various disease conditions, including stroke, neonatal-perinatal cerebral hypoxia, and stroke haemorrhagic transformation [15, 16]. Mechanisms of hypoxia-induced BBB injury that lead to brain edema include aquaporin upregulation, tight junction damage, the accumulation of oxygen free radicals, and glial phagocytosis [17, 18]. The disruption of tight junctions (TJs) is an important cause of BBB injury and brain edema caused by hypoxia [19]. Studies have found that damage to brain microvascular endothelial cells (BMECs), which are the major component of the BBB [20], can cause BBB damage and induce cerebral edema [21]. It has also been demonstrated that cerebrovascular endothelial injury caused by hypoxia is closely related to cerebral edema [22–24]. Since the endothelium is a key structure of the BBB, we speculate that plateau exposure could potentially disrupt the BBB through functional changes to the endothelium.

Endothelial TJs are critical components for maintaining endothelial cell function and BBB integrity. Interendothelial junctions contain complex junctional structures, namely, adherens junctions, tight junctions, and gap junctions, that play pivotal roles in tissue integrity, barrier function, and cell‒cell communication, respectively. [25] Studies have revealed that hypoxia can downregulate the expression of TJ proteins, specifically claudin-5, occludin, and ZO-1 [26–28]. Among the TJ proteins, claudin-5 has been reported to be highly expressed in the endothelial cell and may be involved in regulating BBB permeability [29]. However, the mechanism by which claudin-5 regulates HACE remains unclear.

As a participant in clathrin-independent endocytosis, CAV-1 plays an important role in regulating various cellular processes, including endocytosis, cell growth, differentiation, cholesterol transport, and cellular senescence [30]. CAV-1 is a constitutive structural protein of caveolae in the plasma membrane, and caveolae are a type of lipid raft that maintains the function of endothelial cells [31]. Methyl-β-cyclodextrin (MβCD) is a water-soluble heptasaccharide that acts as a cholesterol scavenger by binding to cholesterol through a hydrophobic core, mainly by disrupting lipid raft structures. Therefore, MβCD can reduce the amount of cholesterol on the membrane and inhibit caveolae-mediated endocytosis [32]. Studies have shown that CAV-1 can increase BBB permeability through transendothelial cell trafficking [33, 34] and by mediating TJ protein endocytosis and translocation [35]. It has been found that hypoxia upregulates CAV-1 expression in endothelial cells and smooth muscle cells [36, 37]. In our previous work, the target genes of NRF1 were screened out by the chromatin immunoprecipitation (ChIP)-seq technique, and it was found that the differentially expressed genes were enriched in the endocytosis pathway [38]. CAV-1 is a necessary participant in caveolin-dependent endocytosis. Additionally, other studies suggest that the hypoxia-induced upregulation of NRF1 in HUVECs has a regulatory effect on plateau exposure-induced blood pressure elevation [39]. We speculate that HH may transcriptionally activate CAV-1 by upregulating NRF1 in BMECs. Thus, the endocytosis and metabolism of the TJ protein claudin-5 is enhanced, TJs are disrupted to increase BBB permeability, and ultimately, HACE is exacerbated.

Therefore, this article aims to explore (1) whether HH exacerbates HACE by damaging endothelial cells, (2) whether CAV-1 mediates the endocytic translocation of claudin-5, resulting in the disruption of tight junctions and BBB permeability, and (3) the possible mechanisms by which hypoxia upregulates NRF1 to regulate CAV-1.

## Materials and methods

### Animals and treatment

Eight-week-old male C57BL/6 mice with an average body weight (BW) of 20 ± 2 g were provided by the Experimental Animal Centre of Nantong University. The mice were allowed to acclimatize to the surrounding environment for 3 d before the experiments began. The mice were maintained in a temperature-controlled room at 26 ± 1 °C with a 12 h light–dark cycle and free access to a standard diet and water. During the HH treatment, the mice were exposed to a simulated altitude of 7600 m (25,000 ft, 282 mmHg) at a 300 m/min velocity in a hypobaric chamber for 24 h. Following this, the mice descended to sea level at the same speed. The mice were immediately anaesthetized and perfused with physiological saline solution (0.9%) to remove the blood. To inhibit caveolae-mediated endocytosis, the mice were injected via the tail vein with MβCD (300 mg/kg) 24 h before HH exposure. After the HH treatment, mice were reinjected with MβCD and immediately anaesthetized and perfused approximately 1 h after circulation.

### Brain water content assay

Brain water content was measured by the wet‒dry weight method [40]. Brain tissue of the mice was collected after the HH treatment and weighed as wet weight. The brain tissue was fully dried in a drying oven at a constant temperature of 100 °C and weighed several times to obtain a stable dry weight (average weighing error < 0.002 g). The percentage of brain water content was calculated as follows:

1 $${\text{Brain}}\;{\text{water}}\;{\text{content}}\; = \left( {{\text{wet}}\;{\text{weight}}\; - {\text{dry}}\;{\text{weight}}} \right)/{\text{wet}}\;{\text{weight}}\; \times \;100\%$$

### BBB permeability assay

Evans blue (EB) staining was performed to evaluate brain vascular leakage. After HH treatment, mice were injected via the tail vein with 4 mL/kg of 2% EB in saline. Approximately 1 h after circulation, the mice were anaesthetized and perfused with physiological saline solution (0.9%) to remove the intravascular dye. Half of the brain was immediately weighed, homogenized in 1 mL of 50% trichloroacetic acid solution, and then centrifuged at 15,000 × g for 30 min. The supernatant was then diluted 1:3 with ethanol, and its absorbance was measured at 630 nm using a microplate reader (Synergy 2™, Bio-Tek, US). The other half of the brain was fixed and sectioned for confocal imaging with a 647 nm laser by a Leica SP8. The mice were also intravenously injected with 40 kD FITC-dextran (D1845, Thermo; 4 mL/kg of 10 mg/mL in saline), which circulated for 15 min. At the end of the circulation period, the mice were promptly euthanized and perfused with physiological saline solution (0.9%) via the left ventricle to remove the blood.

### Cell culture and treatments

Mouse brain microvascular endothelial cells (bEnd.3, Bioleaf Biotech) were maintained in Dulbecco’s modified Eagle’s medium (DMEM, Gibco, US) supplemented with 10% foetal bovine serum (FBS, Sigma, US) at 37 °C in a 5% CO_2_ incubator. Human umbilical vein endothelial cells (HUVECs) were purchased from ScienCell Research Laboratories. The cells were cultured in ECM (1001, ScienCell, US) supplemented with FBS, endothelial cell growth supplement (ECGS), and antibiotic solution. After forming a confluent monolayer, the cells were exposed to hypoxia (94% N_2_, 5% CO_2_, and 1% O_2_) for 24 h. BEnd.3 cells or HUVECs were then incubated with MβCD (5 mM) for 1 h after hypoxia treatment. For the autophagy analysis, a monolayer of cells was incubated with a medium containing 10% FBS, 10 mmol/L 3-methyladenine (3-MA), and 50 nmol/L rapamycin (Rapa) for 2 h. The cells were subsequently exposed to hypoxia and cultured under consistent concentrations of 3-MA and Rapa. [41]

### Transendothelial electrical resistance (TEER) and transcytosis assay

BEnd.3 cells were seeded on 12-transwell inserts (3401, Corning) and cultured to 90% confluence. For the TEER assay, cells were incubated with Hank’s balanced salt solution (HBSS) for 30 min. The total resistance of cells was measured by a Millicell ERS-2 Epithelial Volt-ohm Meter (Merck Millipore). The resistance of HBSS was recorded as the blank resistance. TEER was calculated as follows:

2 $${\text{TEER}}\;\left( {\Omega \cdot{\text{cm}}^{{2}} } \right)\; = \;\left( {{\text{total }}\;{\text{resistance}} - {\text{blank}}\;{\text{resistance}}} \right)\left( \Omega \right) \times {\text{insert}}\;{\text{area}}\left( {{\text{cm}}^{{2}} } \right)$$

Lower resistance indicates damage to the tight junction. For the transcytosis assay, 100 μg/mL 40 kD FITC-dextran (D1845, Thermo) was added to the inserts for 30 min. The medium in the lower plate was then collected and detected by a microplate reader (excitation wavelength 490 nm; emission wavelength 520 nm). The values were converted to FITC-dextran concentrations based on the standard curve.

### Endocytosis assay

After the cells were cultured to a confluence over 90%, the monolayer of either bEnd.3 cells or HUVECs was incubated with 100 μg/mL 40 kD FITC-dextran after hypoxia exposure and then cultured at 37 °C in a 5% CO_2_ incubator for 30 min. The cells were immediately chased with fresh medium for confocal imaging at 488 nm by a Leica SP8.

### Western blot

Cells were lysed in RIPA buffer, and the protein concentration was calculated by bicinchoninic acid assay (BCA). Proteins were isolated through SDS‒PAGE and transferred to PVDF membranes. The membranes were blocked with 5% nonfat dry milk and then incubated overnight at 4 °C with primary antibodies, including anti-NRF1 (CST, 46743S), anti-CAV-1 (CST, 3267), anti-VE-cadherin (Santa Cruz, sc-9989), anti-occludin (Proteintech, 13,409-1-AP), anti-claudin-5 (Thermo, 35-2500), and anti-β-actin (Sigma, A5316). The binding of primary antibodies was visualized with a goat anti-rabbit HRP conjugated secondary antibody (Jason, 115-035-033) or goat anti-mouse HRP conjugated secondary antibody (Jason, 111-035-003).

### Transfection siRNA

Small interfering RNAs (siRNAs) targeting NRF1 (siNRF1) and CAV1 (siCAV1) were synthesized by Suzhou GenePharma Co., Ltd. (Suzhou, China). For transfection, bEnd.3 cells or HUVECs were incubated and cocultured in 12-well culture dishes at 50–60% confluence without antibiotics. siRNA was transfected into bEnd.3 cells or HUVECs using Lipofectamine 2000 (Invitrogen) following the manufacturer’s instructions. After 48 h of transfection, the cells were exposed to hypoxia for 24 h and harvested. Then, the knockdown efficiency was evaluated by Western blotting.

### Transmission electron microscope (TEM)

At the end of the HH treatments, the mouse brains were quickly removed, placed into precooled fixative solution (4% glutaraldehyde; Sigma), and incubated at 4 °C overnight. Samples were dehydrated in a graded ethanol series and embedded in plastic. The sections were then cut at 70–90 nm and stained with 4% uranyl acetate and 0.3% lead citrate. Representative areas from the sections were viewed with HT7700 transmission electron microscope (HITACHI, Japan).

### Real-time PCR

Total RNA was isolated from cells using TRIzol (Invitrogen), and cDNA was generated from 1 μg RNA using the HiScript ®III RT SuperMix kit (Vazyme, R323-01) according to the manufacturer’s instructions. Real-time PCR was performed using the SYBR qPCR Master Mix kit (Roche, USA) at 95 °C for 3 min, followed by 40 cycles of 95 °C for 10 s and 60 °C for 30 s. All primers used for real-time PCR were designed as follows: *Nrf1* forward: 5′ TAT GGC GGA AGT AAT GAA AGA CG 3′, reverse: 5′ CAA CGT AAG CTC TGC CTT GTT 3′; *Cav1* forward: 5′ AGC AAA AGT TGT AGC GCC AG 3′, reverse: 5′ GAC CAC GTC GTC GTT GAG AT 3′; *Actb* forward: 5′ CAT CCG TAA AGA CCT CTA TGC CAA C 3′, reverse: 5′ ATG GAG CCA CCG ATC CAC A 3′. Relative gene expression was calculated using ΔΔCt for normalization to the reference gene.

### Immunofluorescence (IF)

Tissue sections (40 μm) and cultured cells were fixed with 4% paraformaldehyde and permeabilized with 0.3% Triton X-100. The samples were then blocked with 10% donkey serum and probed with anti-CD31 (RD, AF3628), anti-Laminin (Abcam, ab11575), anti-VE-cadherin (Santa Cruz, sc-9989), anti-occludin (Proteintech, 66,378-1-Ig), anti-claudin 5 (Thermo, 35-2500), anti-NRF1 (CST, 46743S), anti-CAV-1 (CST, 3267), anti-ZO-1 (Proteintech, 21,773-1-AP) or anti-LC3 (CST, 12741S) antibodies. The binding of primary antibodies was visualized with either Alexa Fluor 555-conjugated donkey anti-rabbit IgG (Thermo, A31572), Alexa Fluor 647-conjugated donkey anti-mouse IgG (Thermo, A32787), or Alexa Fluor 488-conjugated donkey anti-goat IgG (Abcam, ab150133). The samples were then counterstained with DAPI (Thermo) and imaged using a Leica SP8 confocal microscope.

### Statistical analysis

All data were analysed using GraphPad Prism software version 8.0 (GraphPad Software, San Diego, CA). All sets of continuous data were tested for normality using the Shapiro‒Wilk test, and fewer than 5% of the tests concluded that the set was nonnormal at the 0.05 significance level, confirming that the datasets met the assumption of a normal distribution. Two variants were compared by t test (two-tailed) for independent samples for the results of the NN vs. HH comparison, and comparisons between several groups were performed using ordinary one-way ANOVA. Then, two-way ANOVA followed by Tukey’s multiple comparisons test was used when more than 1 variable was compared. The data are presented as the mean values ± SEMs for in vivo experiments or mean values ± S.D.s for in vitro experiments. Statistical significance was set at *P < 0.05, **P < 0.01, and ***P < 0.001, and n.s indicates no significance.

## Results

### HH exposure induces BBB destruction and brain edema

**Fig. 1:**
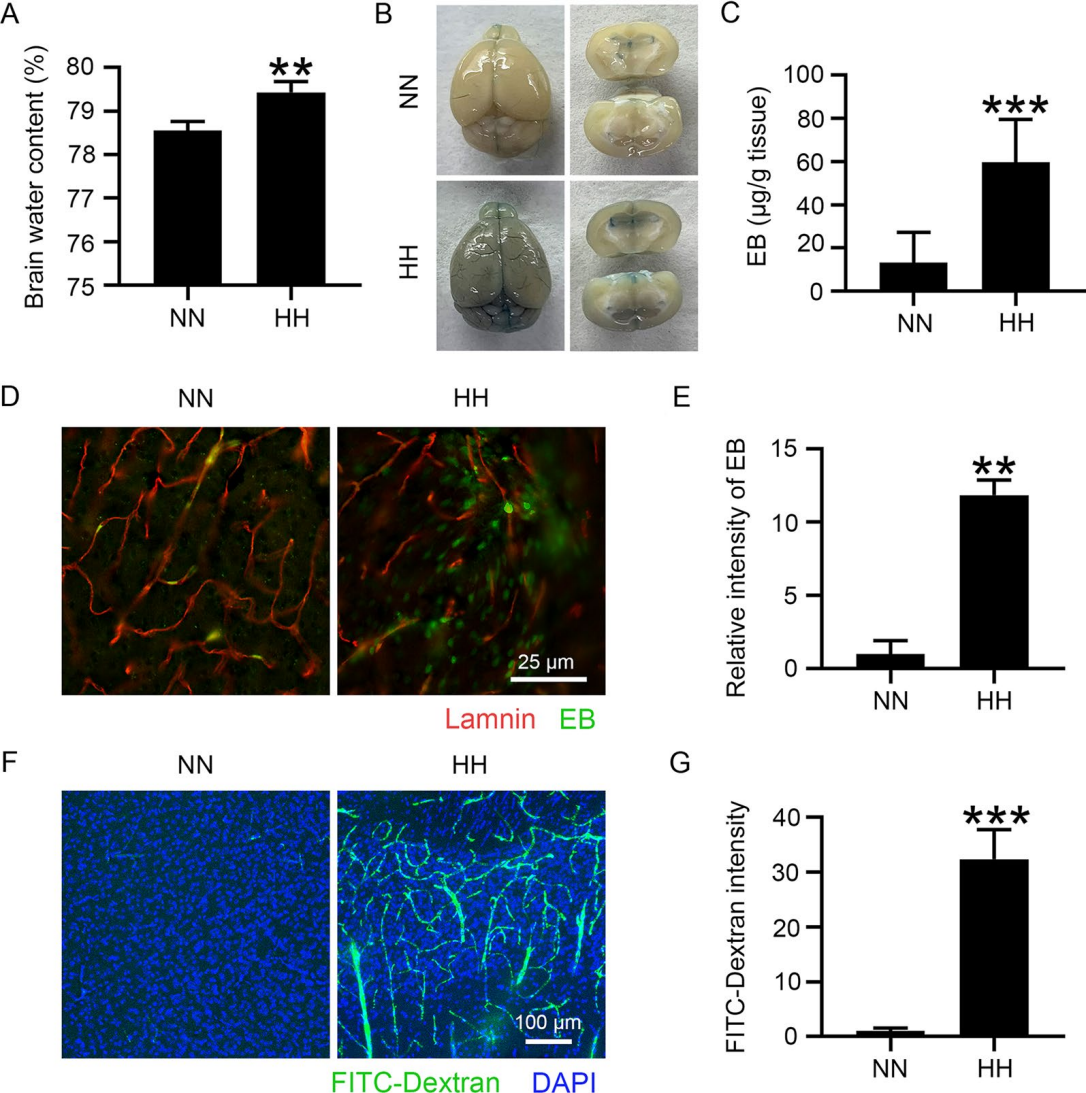
HH induces the disruption of the BBB, triggering brain edema. C57BL/6 mice were exposed to HH (7600 m altitude) for 24 h. **A** Brain water content was measured by the wet and dry weight method. **B** to **E** HH-treated mice were tail-injected with EB, which circulated for 1 h. This was followed by saline perfusion to remove the dye from circulation. The whole brain was observed for EB infiltration into blood vessels and tissues (**B**). The residual amount of EB in tissues was measured by colorimetric assay (**C**). The brain slices were labelled with Laminin by immunofluorescence, and EB distribution in the extravascular tissue was observed (**D**). The fluorescence intensity of Evans blue was calculated (**E**). **F** and **G** HH-treated mice were tail-injected with 40 kD FITC-dextran for 15 min and subsequently perfused with saline to remove it from circulation. Brain slices were labelled with DAPI, and dextran distribution in the cortex was observed (**F**). The fluorescence intensity of dextran was measured (**G**) (*P < 0.05, **P < 0.01 and ***P < 0.001, n = 10)

To construct the HACE animal model, C57BL/6 mice were exposed to HH (7600 m above sea level) for 24 h. The brain water content of HH-treated mice was significantly increased (Fig. 1A), demonstrating the success of the model. To observe BBB integrity, EB was injected via the tail vein and allowed to circulate for 1 h. The mice were then perfused with saline to remove the intravascular EB. The results demonstrated that the residual EB content in the mouse brains was higher after HH exposure (Fig. 1B and C). To further confirm whether the increased residual EB in the HH group was due to its infiltration into the brain tissue, we observed whether EB leakage occurred around the cerebral blood vessels. Figure 1D and E reveal that distinct EB signals were observed in cerebral perivascular tissue, suggesting BBB damage. We further explored the integrity of the BBB by exogenously injecting FITC-dextran. A bright green signal appeared around the blood vessels in the HH group, suggesting that BBB permeability increased after HH exposure (Fig. 1F and G). The above results demonstrated that BBB permeability significantly increased in the HACE mouse model when induced by HH exposure.

### Hypoxia impairs BBB integrity by reducing endothelial tight junction protein levels

**Fig. 2:**
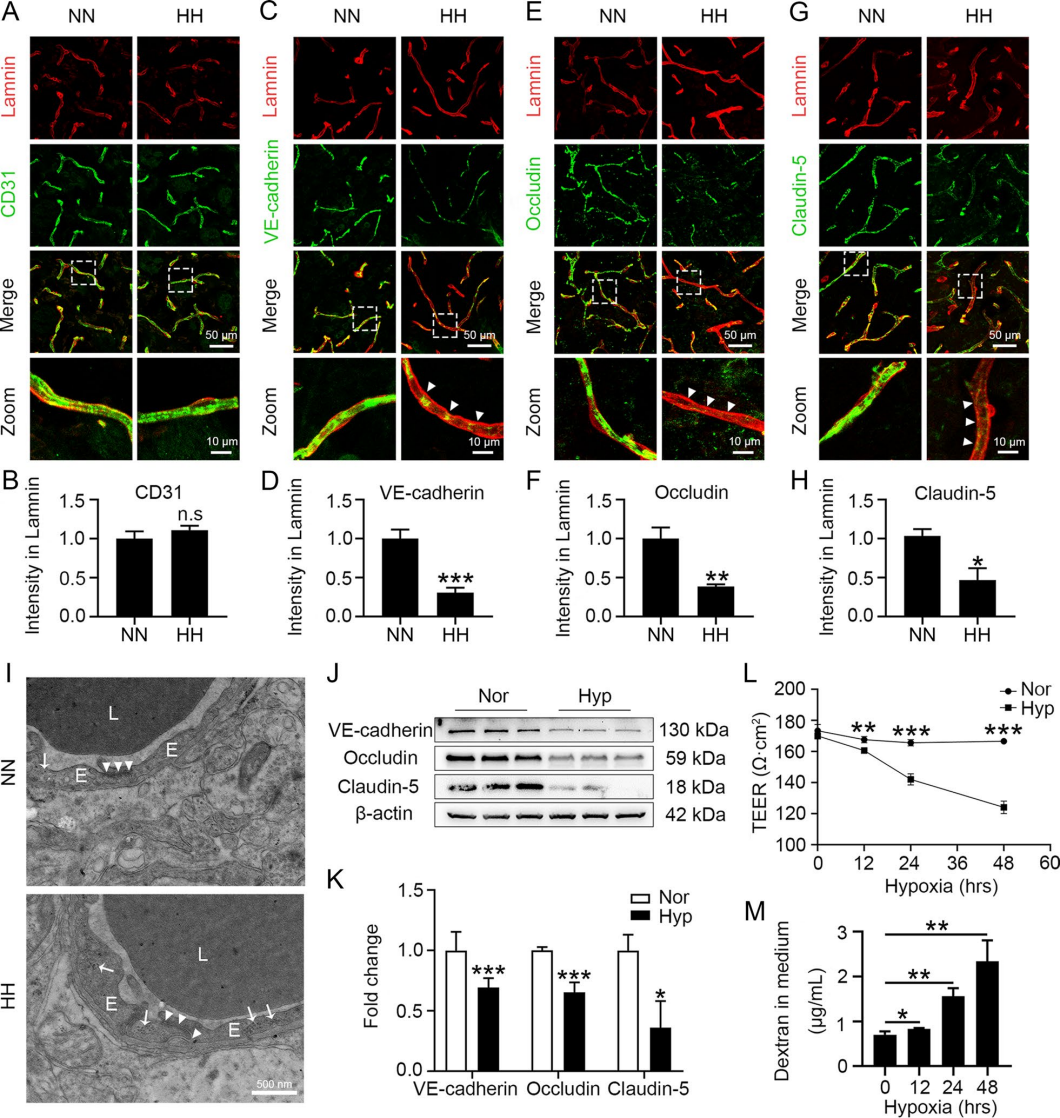
Hypoxia enhanced the destruction of TJs both in vivo and in vitro. **A** to **I** C57BL/6 mice were exposed to HH (7600 m above sea level) for 24 h. Brain slices were labelled with immunofluorescence stains for Laminin. Then, they were colabelled for CD31 (**A**), VE-cadherin (**C**), occludin (**E**) or claudin-5 (**G**), and the fluorescence intensity of CD31 (**B**), VE-cadherin (**D**), occludin (**F**) or claudin-5 (**H**) in blood vessels was counted separately (*P < 0.05, **P < 0.01 and ***P < 0.001, n = 10). Electron microscope observation of vascular tight junction ultrastructure (**I**) (E, endothelial cells; L, vascular lumen; TJ, short arrows; endothelial vesicles, long arrows. n = 4). **J** to **M** bEnd.3 cells were exposed to 1% O_2_ for 24 h or the indicated time. The protein levels of VE-cadherin, occludin, and claudin-5 were determined by Western blot (**J**) and quantified (**K**) (* *P* < 0.05, and *** *P* < 0.001). The endothelial permeability was detected by TEER (**L**) and leakage of 40 kD FITC-dextran (**M**) (*P < 0.05, **P < 0.01 and ***P < 0.001)

We first focused on endothelial cell integrity to demonstrate whether the increased BBB permeability induced by HH exposure was associated with BMECs. As displayed in Fig. 2A and B, HH did not trigger the shedding or reduction of endothelial cells. As endothelial TJs are critical for BBB integrity, we further examined whether endothelial TJs were impaired. We found that the continuity of tight junction proteins, including VE-cadherin, occludin, and claudin-5, was significantly disrupted, and the protein levels were significantly reduced after HH exposure (Fig. 2C to H). Furthermore, the ultrastructure indicated a marked swelling of the endothelial cells, disruption of tight junctions, and many vesicle-like structures visible within the endothelium (Fig. 2I), suggesting that HH exposure triggered the disruption of endothelial tight junction structures. To investigate whether hypoxia is a key reason for the decrease in endothelial tight junction proteins, bEnd.3 cells were treated with 1% O_2_ for 24 h, and the expression level of HIF-1a was detected to observe the effect of hypoxia (Additional file 1: Fig. S1A). Figure 2J and K demonstrate that hypoxia led to a significant downregulation of endothelial connexin protein levels (VE-cadherin, occludin, and claudin-5). We found that hypoxia treatment significantly decreased endothelial transmembrane resistance (Fig. 2L), which suggests that hypoxia induced an increase in endothelial permeability. Furthermore, hypoxia enhanced the transmembrane transport of dextran as endothelial permeability increased (Fig. 2M), which is consistent with the in vivo results. Overall, the above data suggest that hypoxia increases BBB permeability by downregulating tight junction protein levels and disrupting endothelial TJs.

### CAV-1-mediated endocytosis promotes hypoxia-upregulated endothelial permeability and triggers HACE

**Fig. 3:**
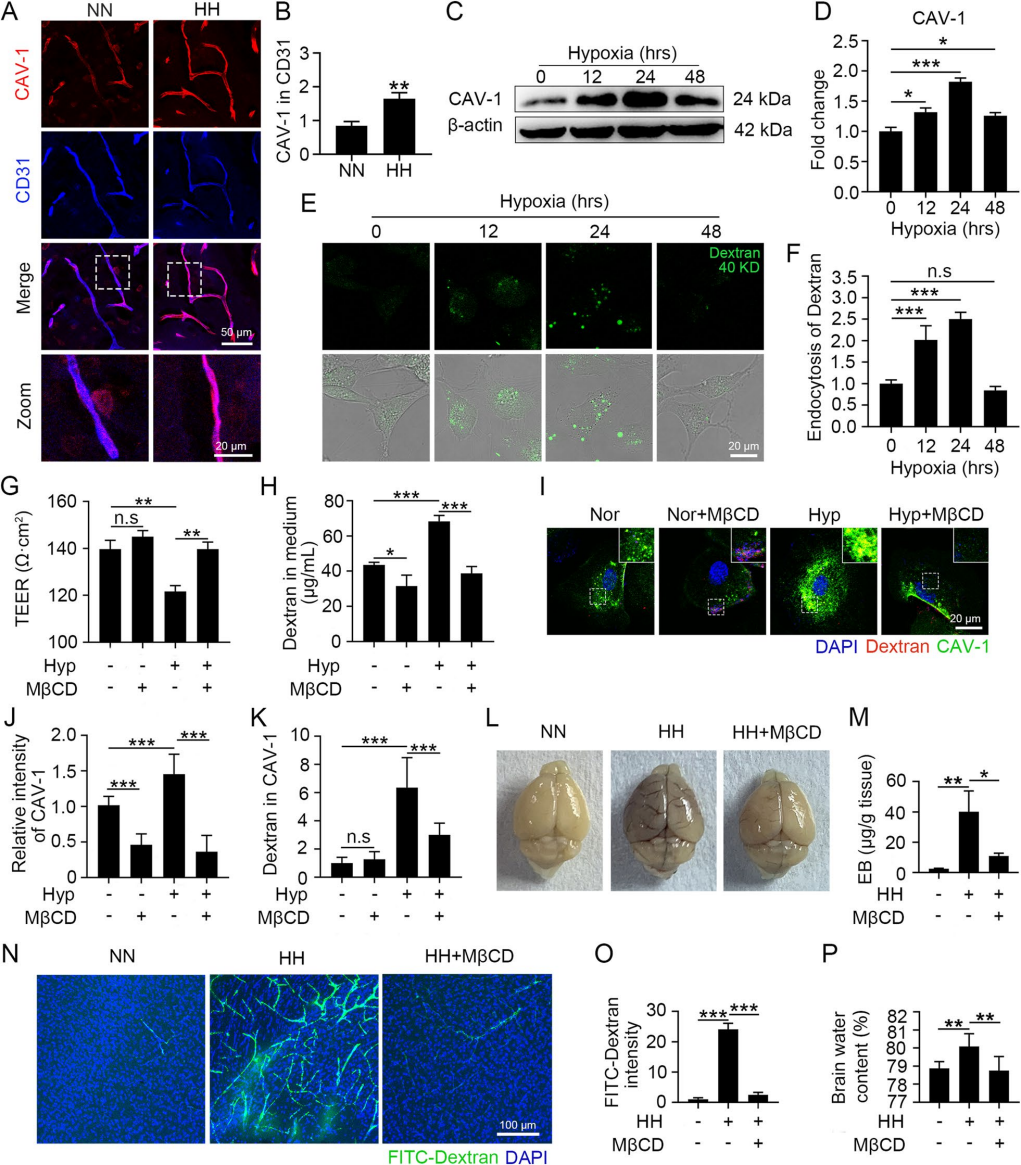
Hypoxia enhanced endothelial permeability through the upregulation of CAV-1-mediated endocytosis and triggered HACE. **A** and **B** C57BL/6 mice were exposed to HH (7600 m altitude) for 24 h. Brain slices were colabelled with CD31 and CAV-1 (**A**), and the fluorescence intensity of CAV-1 in the endothelium was counted (**B**) (**P < 0.01, n = 10). **C** to **F** bEnd.3 cells were exposed to 1% O_2_. The CAV-1 protein levels were detected by Western blot (**C**) and quantified (**D**). FITC-dextran was incubated with hypoxia-treated cells for 30 min (**E**), and the level of endocytosed dextran was calculated by counting the intensity in cells (**F**) (* *P* < 0.05 and *** *P* < 0.001). **G** to **K** bEnd.3 cells were treated with hypoxia for 24 h and then incubated with 5 mM MβCD for 1 h. After 30 min of FITC-dextran treatment, cell permeability was detected by TEER (**G**) and leakage of 40 kD FITC-dextran (**H**). CAV-1 was labelled by immunofluorescence (**I**), the intensity of CAV-1 (**J**) and dextran colocalized with CAV-1 (**K**) were counted (*P < 0.05, **P < 0.01 and ***P < 0.001). **L** to **P** C57BL/6 mice injected with 300 mg/kg MβCD were exposed to HH for 24 h and then reinjected with MβCD, which circulated for 1 h. After injecting EB via the tail vein for 1 h, the infiltration of EB into the blood vessels and tissues was observed (**L**) and the residual EB content in the tissues was quantified by colorimetry (**M**). Mice were injected with 40 kD FITC-dextran through the tail vein for 15 min, and the dextran distribution in the cortex was observed (**N**). The dextran fluorescence intensity was counted (**O**). The brain water content was measured by the dry and wet weight method (**P**) (*P < 0.05, **P < 0.01 and ***P < 0.001, n = 7)

To investigate the role of CAV-1 in HH-induced TJ downregulation, we detected the levels of CAV-1 in the BMECs of HH-exposed mice in situ. CAV-1 protein levels were significantly upregulated in BMECs following HH treatment (Fig. 3A and B). Consistently, CAV-1 expression levels were increased in hypoxia-treated bEnd.3 cells, and this increase peaked at 24 h (Fig. 3C and D). These findings suggest that hypoxia upregulated the expression of CAV-1, which led to HH-induced BBB injury. In addition, we verified that the upregulation of CAV-1 expression enhanced the endocytic capacity of endothelial cells. The upregulation of the endocytic capacity of hypoxia-treated bEnd.3 cells was consistent with the change in the trend of CAV-1 expression, with the highest endocytic capacity at 24 h (Fig. 3E and F). Therefore, we conducted hypoxia treatments for 24 h as the experimental condition during subsequent experiments. To demonstrate that hypoxia-induced changes in endothelial permeability are dependent on CAV-1, we treated hypoxic endothelial cells with MβCD, a specific blocker of CAV-1. We found that blocking CAV-1 significantly reversed the hypoxia-induced decrease in endothelial cell transmembrane resistance and the increase in dextran transmembrane transport (Fig. 3G and H). Moreover, it was demonstrated that blocking CAV-1 significantly downregulated the expression level of CAV-1 (Fig. 3I and J) and inhibited CAV-1-mediated endocytosis (Fig. 3K). This suggests that the hypoxia-induced enhancement of endothelial permeability depends on the upregulation of CAV-1 and increased endocytosis. To investigate the role of CAV-1 in HH-induced BBB injury and cerebral edema, we pretreated HH-treated mice with MβCD. MβCD significantly improved HH-induced EB residues (Fig. 3L and M) and FITC-dextran leakage (Fig. 3N and O). This reduced the HH-induced increases in endothelial permeability, suggesting that blocking CAV-1-mediated endocytosis is effective in ameliorating HH-induced BBB injury. Furthermore, MβCD significantly ameliorated the HH-induced increase in water content, demonstrating that blocking CAV-1 effectively prevented HACE (Fig. 3P).

### CAV-1 promotes hypoxia-injured endothelial TJs by mediating the internalization of claudin-5

**Fig. 4:**
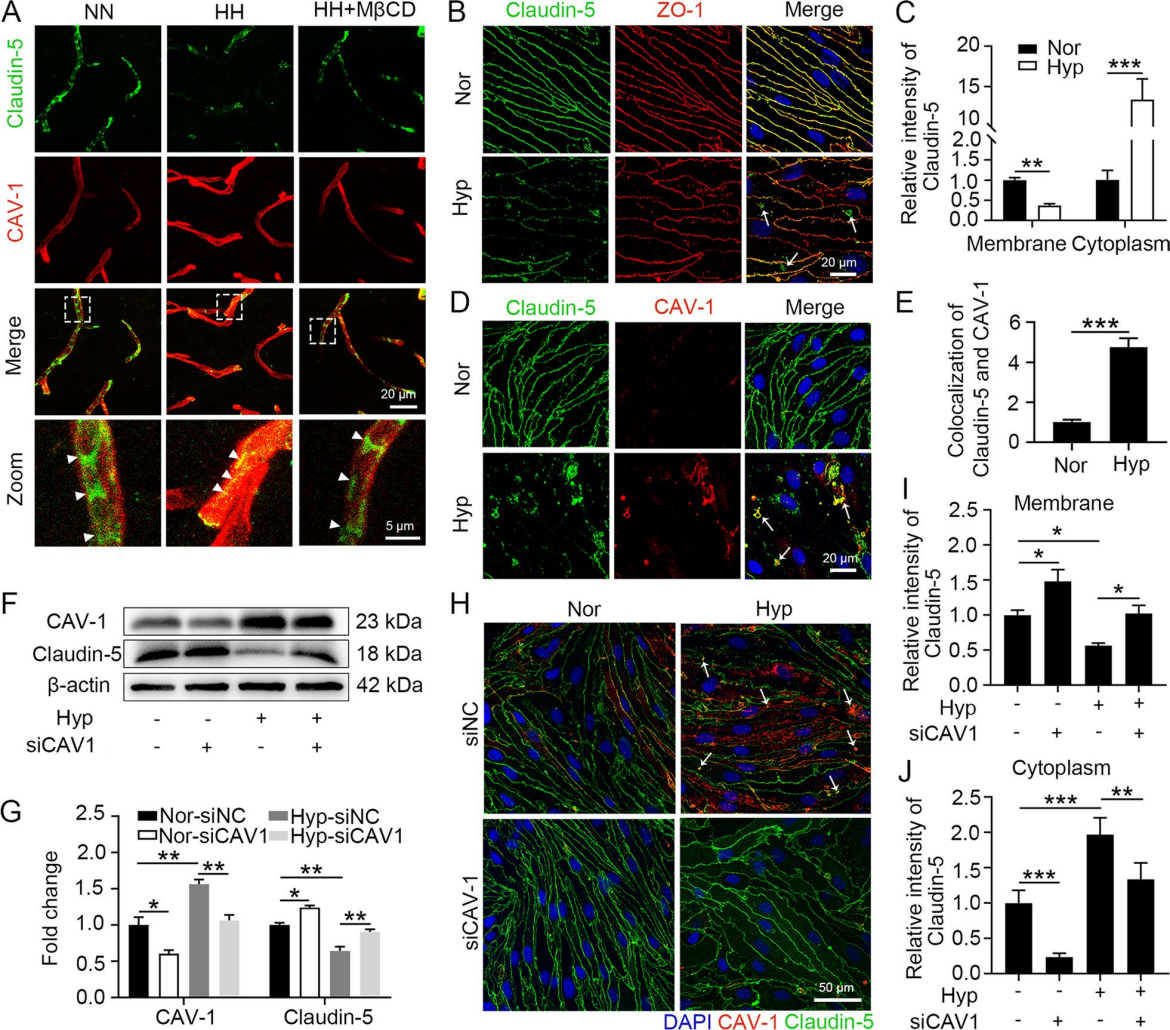
Hypoxia promoted the internalization of claudin-5 in a CAV-1 upregulation-dependent manner. **A** C57BL/6 mice injected with 300 mg/kg MβCD were exposed to HH for 24 h and then reinjected with MβCD, which cycled for 1 h. Brain slices were colabelled with claudin-5 and CAV-1 antibodies to observe the colocalization of CAV-1 and claudin-5 (n = 10). **B** to **E** bEnd.3 cells were exposed to 1% O_2_ for 24 h. Claudin-5 and ZO-1 antibodies were labelled by immunofluorescence (**B**) to quantify the fluorescence intensity of claudin-5 on the cell membrane and in the cytoplasm (**C**). Claudin-5 and CAV-1 antibodies were labelled by immunofluorescence (**D**) to calculate the colocalization of CAV-1 and claudin-5 (**E**) (** *P* < 0.01 and *** *P* < 0.001). **F** to **J** After transfection with siCAV1 for 48 h, bEnd.3 cells were exposed to 1% O_2_ for 24 h. The CAV-1 and claudin-5 protein levels were detected by Western blot (**F**) and quantified (**G**). CAV-1 and claudin-5 antibodies were labelled by immunofluorescence assay (**H**) to quantify the fluorescence intensity of claudin-5 on the cell membrane (**I**) or in the cytoplasm (**J**) (*P < 0.05, **P < 0.01 and ***P < 0.001)

To further investigate the mechanisms by which CAV-1 induces changes in endothelial cell permeability, we labelled CAV-1 and claudin-5 proteins in mouse brain tissue. CAV-1 fluorescence intensity was significantly enhanced, but claudin-5 fluorescence intensity was significantly reduced in the BMECs of HH-treated mice (Fig. 4A). In contrast, MβCD effectively reversed the fluorescence intensity changes in CAV-1 and claudin-5, suggesting that CAV-1-mediated endocytosis regulates claudin-5 protein levels. Moreover, the colocalization of CAV-1 with claudin-5 was increased in HH-treated mouse brains, and MβCD significantly reduced this colocalization effect. These findings suggest that CAV-1 reduces the protein levels of claudin-5 and impairs BBB integrity by mediating claudin-5 endocytosis. Hypoxia-induced intracellular translocation of claudin-5 was demonstrated by labelling claudin-5 associated with hypoxia-induced bEnd.3 tight junction proteins (Fig. 4B and C). The colocalization results of CAV-1 and claudin-5 after hypoxia treatment also suggested that the intracellular translocation of claudin-5 was caused by CAV-1-mediated endocytosis (Fig. 4D and E). To demonstrate the relationship between CAV-1 and claudin-5 internalization and downregulation, we modulated CAV-1 levels in hypoxia-treated bEnd.3 cells. CAV-1 interference resulted in a significant upregulation of claudin-5 protein levels in both normoxic and hypoxic endothelial cells, demonstrating that hypoxia-induced downregulation of claudin-5 expression was dependent on CAV-1 (Fig. 4F and G). Occludin expression was also significantly upregulated after blocking CAV-1 (Additional file 1: Fig. S1B and C). The results in Fig. 4H to J also show a significant decrease in intracellular content that was accompanied by the upregulation of claudin-5 membrane expression upon interference with CAV-1. This finding further highlights that hypoxia induces the internalization and downregulation of claudin-5 through CAV-1-mediated endocytosis.

### Internalized claudin-5 is metabolized by autophagy, resulting in reduced claudin-5 content under hypoxic conditions

**Fig. 5:**
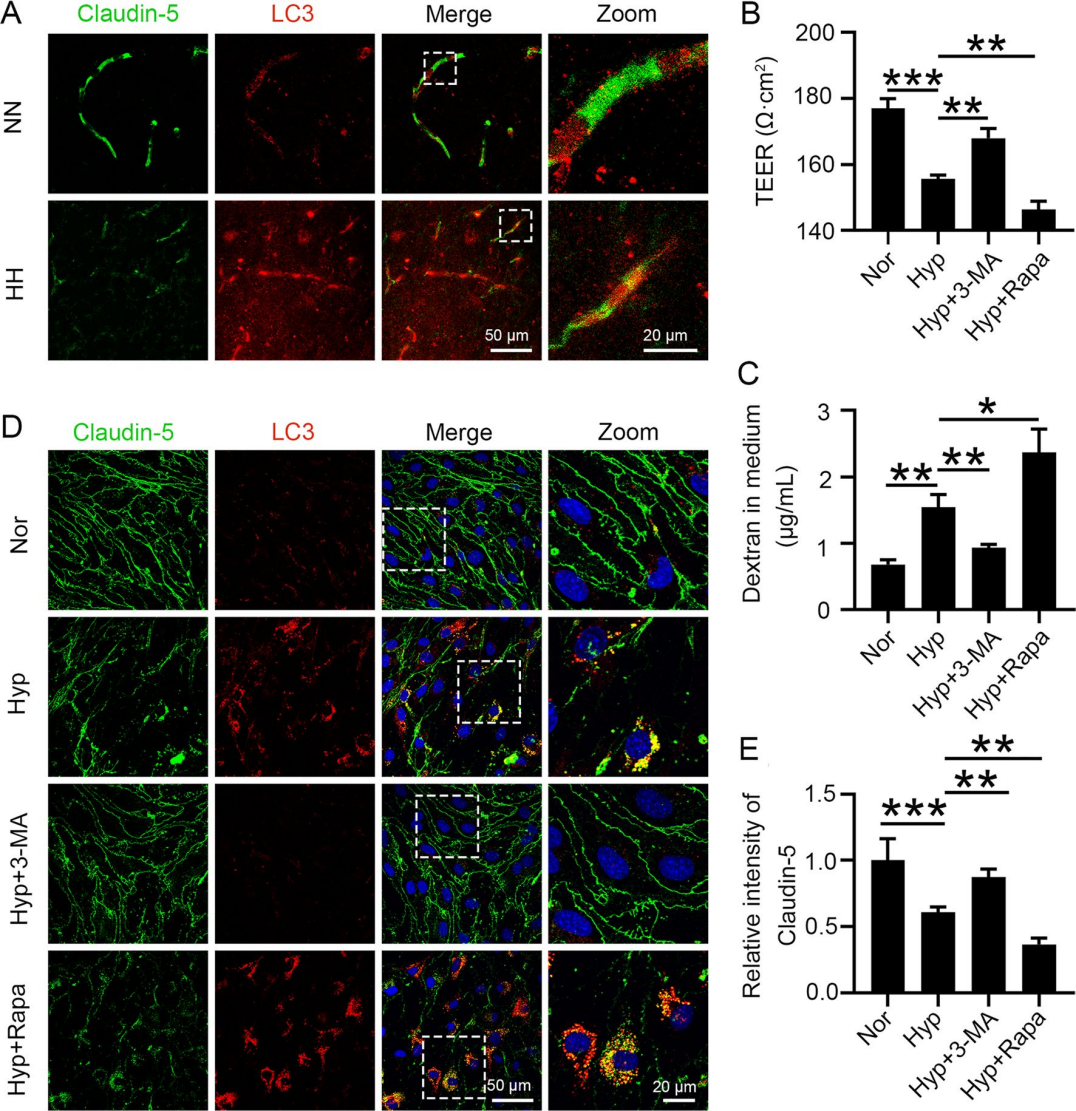
Autophagy participates in the degradation of aggregated claudin-5 in the cytosol. **A** C57BL/6 mice were exposed to HH (7600 m above sea level) for 24 h. Brain slices were colabelled with claudin-5 and LC3 antibodies to observe the colocalization of claudin-5 and LC3 (n = 10). **B** to **E** bEnd.3 cells were pretreated with 50 nmol/L Rapa or 10 mmol/L 3-MA for 2 h, followed by hypoxia treatment for 24 h. Cell permeability was determined by TEER (**B**) and leakage of 40 kD FITC-dextran (**C**) to analyse the effect of autophagy on endothelial barrier function. Claudin-5 and LC3 antibodies were labelled by immunofluorescence (**D**) to count the fluorescence intensity of claudin-5 (**E**) (*P < 0.05, **P < 0.01 and ***P < 0.001)

To explore the pathways of internalized claudin-5 metabolism, we labelled claudin-5 and LC3 in HH-treated mouse brain vessels. We found a significant increase in the colocalization of claudin-5 with LC3 after HH treatment (Fig. 5A). This finding suggests that hypoxia may downregulate claudin-5 levels through autophagic metabolism and increase endothelial permeability. Therefore, we regulated the autophagy levels by administering the autophagy inhibitor 3-MA or the inducer Rapa to hypoxic bEnd.3 cells. 3-MA effectively inhibited the hypoxia-induced downregulation of transmembrane resistance. Additionally, Rapa further exacerbated the decrease in transmembrane resistance, indicating that elevated levels of autophagy could exacerbate hypoxia-induced endothelial tight junction damage (Fig. 5B). Moreover, 3-MA inhibited the hypoxia-mediated transmembrane transport of dextran, and Rapa facilitated this process. These findings demonstrate that the upregulation of autophagy enhances hypoxia-induced endothelial permeabilization (Fig. 5C). Furthermore, we found that 3-MA effectively ameliorated the hypoxia-induced downregulation of claudin-5 and that Rapa exacerbated the hypoxia-induced loss of claudin-5 (Fig. 5D and E). The above findings provide ample evidence that the decline in endothelial claudin-5 levels under hypoxia is dependent on autophagic metabolism.

### Hypoxia induces the upregulation of CAV-1 expression by upregulating the level of NRF1

**Fig. 6:**
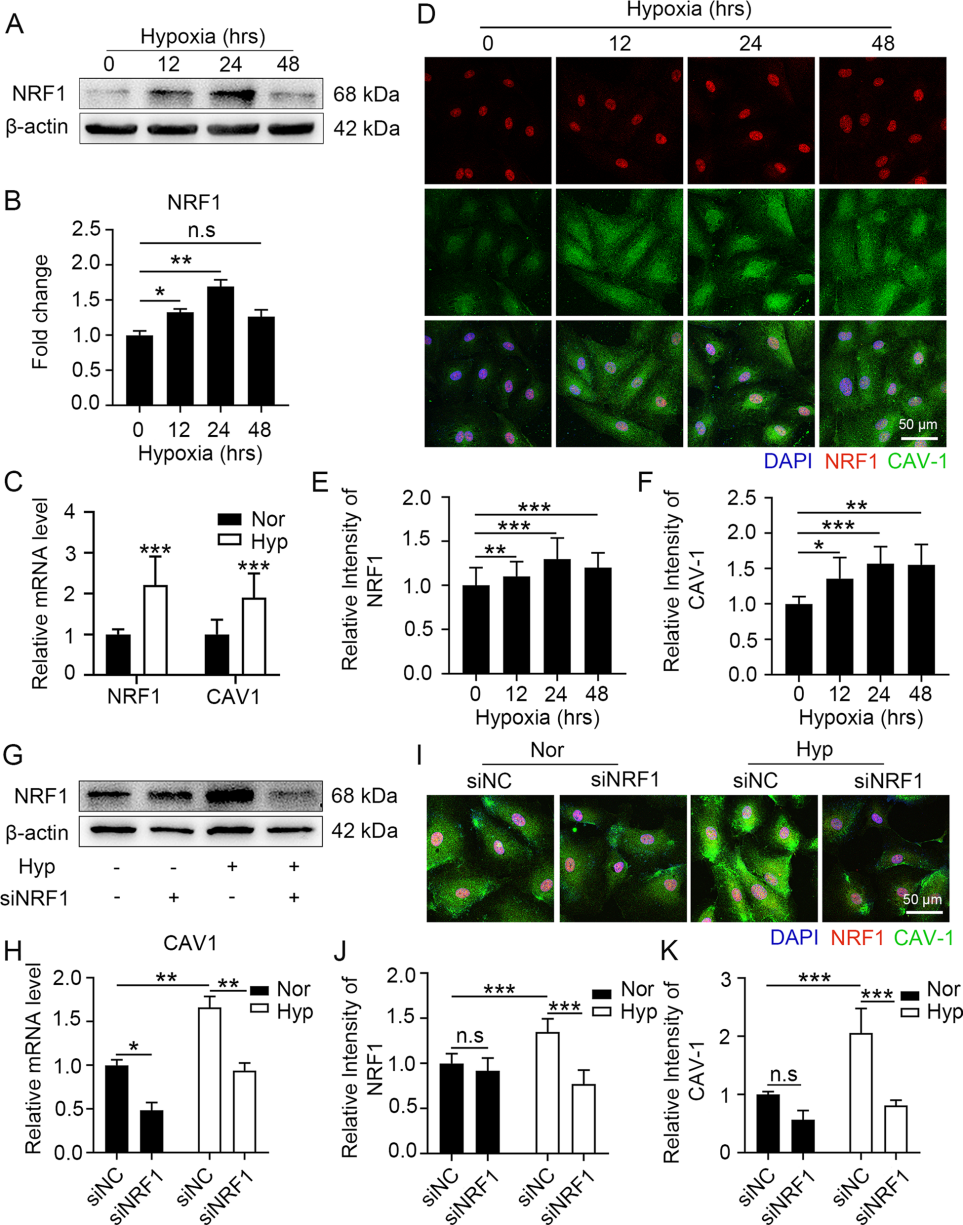
NRF1 upregulated the expression of CAV-1 under hypoxic conditions. **A** to **C** bEnd.3 cells were exposed to 1% O_2_ for 24 h or the indicated times. NRF1 protein levels were detected by Western blot (**A**) and quantified (**B**). The mRNA levels of *Nrf1* and *Cav1* were determined by real-time PCR (**C**) (* *P* < 0.05, ** *P* < 0.01 and *** *P* < 0.001). **D** to **F** HUVECs were exposed to 1% O_2_ for the indicated times. NRF1 and CAV-1 antibodies were labelled by immunofluorescence (**D**) to count the intensity of NRF1 (**E**) and CAV-1 (**F**) (* *P* < 0.05, ** *P* < 0.01 and *** *P* < 0.001). (**G** and **H**) After transfection with siNRF1 for 48 h, bEnd.3 cells were exposed to 1% O_2_ for 24 h. The NRF1 protein levels were detected by Western blot (**G**); *Cav1* mRNA levels were determined by real-time PCR (**H**) (*P < 0.05 and **P < 0.01). (**I** to** K**) After transfection with siNRF1 for 48 h, HUVECs were exposed to 1% O_2_ for 24 h. NRF1 and CAV-1 antibodies were labelled by immunofluorescence (**I**) to count the intensity of NRF1 (**J**) and CAV-1 (**K**) (*** *P* < 0.001)

To further explore the mechanism by which CAV-1 expression is upregulated in endothelial cells under hypoxic conditions, we examined the effect of hypoxia treatment on the protein level of the transcription factor NRF1. NRF1 was significantly upregulated after hypoxia treatment, reaching a peak at 24 h. These findings are consistent with the changes in CAV-1 (Fig. 6A and B). We further examined the alterations of *Nrf1* and *Cav1* mRNA under hypoxia and found that the mRNA levels of *Nrf1* and *Cav1* were upregulated synchronously after 24 h of hypoxia treatment (Fig. 6C). The above results suggest that the transcriptional upregulation of CAV-1 was possibly associated with the upregulation of NRF1. Therefore, we further investigated whether the nuclear translocation of NRF1 was altered by hypoxia. The nuclear translocation of NRF1 was significantly increased after hypoxia, and this was accompanied by the upregulation of CAV-1 levels. These findings suggest that hypoxia regulates CAV-1 expression by activating the nuclear translocation of NRF1 (Fig. 6D to F). To demonstrate that the hypoxia-induced upregulation of CAV-1 depends on NRF1 activation, we interfered with NRF1 expression in hypoxic endothelial cells (Fig. 6G). NRF1 interference significantly downregulated the CAV-1 transcript level in normoxia- or hypoxia-treated bEnd.3 cells (Fig. 6H). Interfering with NRF1 significantly inhibited the upregulation of CAV-1 protein levels by hypoxia (Fig. 6I to K). The above results demonstrate that hypoxia induces the upregulation of CAV-1 expression through the upregulation of NRF1.

## Discussion

**Fig. 7:**
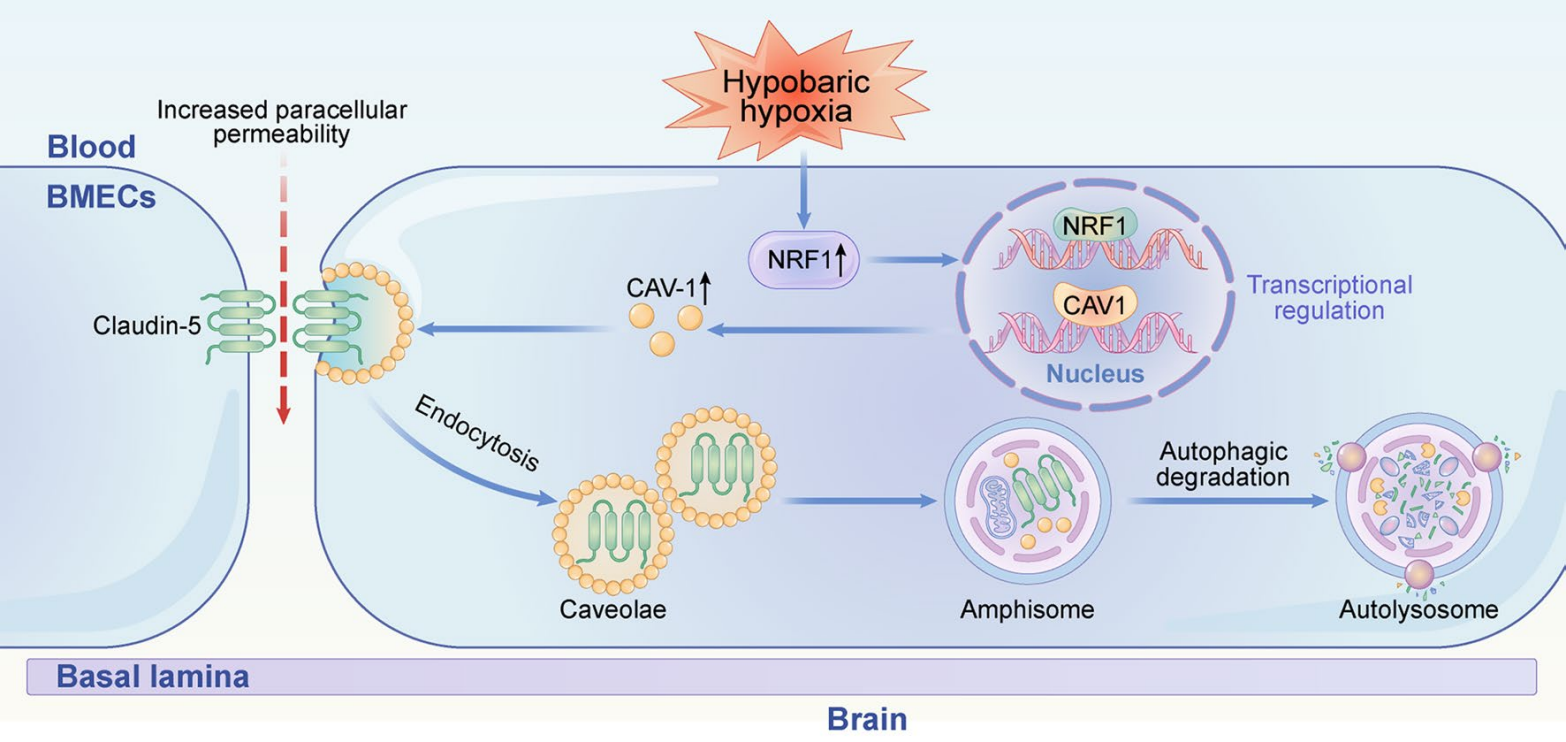
A proposed model of the role of CAV-1 in disrupting the integrity of the BBB under HH. Hypoxia treatment in cerebrovascular endothelial cells upregulates CAV-1 transcription by activating NRF1, thereby inducing a redistribution of the membranous claudin-5. Claudin-5 is further endocytosed by CAV-1-composed caveolae, impairing the integrity and permeability of the BBB. Moreover, endothelial autophagy mediates the clearance of aggregated claudin-5 in the cytosol, leading to BBB disruption and triggering HACE

This study investigated the mechanisms involved in hypoxia-induced endothelial cell dysfunction and the pathological impact of endothelial cell dysfunction on HACE. We aimed to investigate whether CAV-1 is involved in HACE progression and its underlying mechanism in this process. The main experimental results are summarized as follows: (1) HH exposure induces BBB disruption and brain edema; (2) hypoxia causes endothelial damage by reducing endothelial tight junction protein levels and then disrupts BBB integrity; (3) CAV-1-mediated endocytosis under hypoxia increases endothelial cell permeability and promotes HACE; (4) CAV-1 mediates endothelial membrane claudin-5 internalization and translocation, impairing interendothelial junctions; (5) internalization of claudin-5 is metabolized by autophagy, thereby increasing cell permeability and impairing the endothelial barrier; and (6) hypoxia upregulates the transcription of its downstream target gene CAV-1 by inducing the nuclear translocation of NRF1. All these results support our hypothesis that hypoxia upregulates CAV-1 transcription by activating NRF1 in endothelial cells, thereby inducing Claudin-5 internalization and autophagic degradation. These effects lead to BBB disruption and trigger HACE (Fig. 7). Therefore, CAV-1 may become a potential therapy target for HACE.

Significant vascular leakage near the corpus callosum and diffusion of microleakage in the mouse cerebral cortex were observed in our HACE model, which is consistent with the MRI findings in HACE patients [9, 10]. The degradation of endothelial cell TJs can lead to the extravasation of plasma proteins and water, triggering vasogenic edema. The complete degradation of TJs can eventually lead to the disruption of endothelial cell continuity, disintegration of the vascular structure, and leakage of red blood cells into brain tissue, ultimately leading to microhemorrhage [42]. These findings suggest that tight junction proteins play a critical role in regulating vascular endothelial cell permeability and maintaining vascular structural integrity. The hypoxia-induced downregulation of tight junction protein complexes causing increased capillary permeability has been reported as the main pathological change leading to vascular leakage [43]. Drugs such as astragaloside and acetyl-11-keto-β-boswellic acid (AKBA) have been revealed to significantly improve the symptoms of cerebral edema by reducing brain endothelial cell permeability [44, 45]. Therefore, it is currently believed that improving endothelial cell tight junction disruption and reducing brain endothelial cell permeability may serve as effective therapeutic targets for reducing brain edema. We then assessed the changes in the expression levels of TJ proteins, such as VE-cadherin, occludin, and claudin-5, in vascular endothelial cells after HH treatment for 24 h. Our results suggest that the connexins (VE-cadherin, occludin, and claudin-5) are essential for endothelial cell barrier function, as HH exposure reduced the expression levels of these proteins to approximately half of the control levels. To further assess the impact of changes in endothelial TJs on brain endothelial barrier function, we measured changes in TEER and transmembrane transport of dextran in vitro. Our current study shows that the high endothelial cell permeability reflected by reduced TEER and increased transmembrane transport of dextran is consistent with the reduced expression levels of the three connexins. Therefore, these findings may explain, at least in part, the pathophysiology of vascular hyperpermeability in HACE. In this study, the hypoxia-induced impairment of endothelial cell functions was demonstrated both in vivo and in vitro. The findings demonstrate that hypoxia-induced changes in endothelial permeability can contribute to brain edema and that endothelial cells play a key role in HACE pathogenesis.

The severity of brain edema due to various types of brain injury is closely linked to CAV-1 expression. In rats, cortical cold injury was associated with increased CAV-1 expression, and the phosphorylation of CAV-1 was critical for the transcytosis of proteins across the brain endothelium. This leads to BBB decomposition and brain edema after brain injury [46]. CAV-1 is mainly upregulated in endothelial cells within the perihaematomal area of intracerebral haemorrhage. CAV-1 knockout can reduce brain injury volume, alleviate neurological deficits, reduce the activity of matrix metalloproteinase-9, and significantly improve brain edema [47]. Our study found that the hypoxia-induced upregulation of CAV-1 expression in endothelial cells in both in vivo and in vitro studies resulted in enhanced endothelial cell endocytosis. Subsequently, blocking CAV-1 significantly reversed the hypoxia-induced disruption of tight junction proteins and reduced endothelial cell permeability. Moreover, a significant reduction in cerebrovascular leakage and an improvement in brain edema were also observed in HH-exposed mice pretreated with MβCD. Here, we discovered for the first time that CAV-1 has the potential to disrupt the BBB to promote HACE. It has also been found that CAV-1 exhibits positive effects during specific brain injuries. Cerebral infarct volume was significantly upregulated in CAV-1 KO mice compared with WT mice following focal cerebral ischaemia/reperfusion (I/R) injury in the mouse brain [48]. These findings indicate that CAV-1 exhibits different responses during brain injury. This effect is mainly due to its functional association with various molecules, especially signal transduction and endocytosis.

Claudin-5 is regarded as a major tight junction (TJ) protein and is the key protein responsible for maintaining barrier integrity and regulating BBB permeability [29]. In the present study, we found that CAV-1 increased the permeability of BMECs to induce brain edema by mediating the endocytic translocation of claudin-5. This further confirms that regulating transmembrane tight junction protein endocytosis is the main mechanism by which BBB permeability is regulated. We also found significant colocalization of the upregulated LC3B and the aggregated claudin-5. This finding suggests that autophagy may be activated under hypoxia and is involved in hypoxia-induced BBB damage through the degradation of intracytoplasmic translocated claudin-5. Our study suggests that claudin-5 acts as a sensitive marker of hypoxic endothelial injury in HACE and possibility regulates endothelial autophagy. Notably, tight intercellular junctions were prevalent in various epithelial and endothelial cells and in capillary bile ducts and renal tubules in vertebrates, and our experiments were conducted in a simulated hypoxic environment. Therefore, other diseases associated with hypoxia, such as diseases of the nervous system, digestive system, urinary system, and glial lymphatic system, can also draw on this mechanism. This concept may warrant further research in related fields and is subject to further verification.

CAV-1 not only mediates claudin-5 endocytosis but also affects occludin expression in the cerebrovascular endothelium [49]. Occludin can induce CAV-1 accumulation in the cell membrane due to excessive cleavage. It can also promote CAV-1-mediated endocytosis, leading to the lysosomal degradation of other tight junction proteins [50]. In this study, hypoxia treatment induced a significant decrease in occludin expression both in vivo and in vitro. After blocking CAV-1, occludin expression was significantly upregulated. Thus, we speculate that the internalization of occludin in the cytoplasm is related to the degradation of claudin-5 by autolysosomes, which requires further investigation and verification.

In our previous study, we found that CAV-1 and AP2B1 may be downstream target genes of NRF1 through ChIP-seq screening and initially explored the role of CAV-1 in HACE [38]. In this paper, we further studied the specific mechanism by which CAV-1 mediates endocytosis in the occurrence and development of HACE. Furthermore, cytotoxic edema and vasogenic edema are key factors in the development of edema in the central nervous system [42]. MRI data from HACE patients suggest that HACE formation is influenced by vasogenic and cytotoxic edema [9, 10]. In our previous work, we mainly studied the damage to the BBB by hyperactivated microglia and explained the pathogenesis of HACE from the perspective of cytotoxic edema. In this study, we mainly introduced the related pathogenesis of endothelial cell dysfunction to accelerate the progression of HACE from the perspective of vasogenic edema. Therefore, our study further refines the pathogenesis of HACE through two aspects of vasogenic edema and cytotoxic edema.

Studies have demonstrated that NRF1 can regulate autophagy by activating the transcriptional regulation of the promoters of ATG5 and ATG7 [51]. NRF1 may also participate in mitochondrial quality control by regulating PINK1/Parkin-mediated mitochondrial phagocytosis [52]. NRF1 could be involved in regulating autophagy to maintain normal cellular physiological functions and contribute to cellular adaptations to various stresses [53]. Our results showed that the NRF1 levels and autophagy levels were significantly upregulated under hypoxic conditions. Therefore, we hypothesize that NRF1 may be extensively involved in regulating autophagy through multiple pathways. This study found that NRF1 may be involved in hypoxia-induced BBB damage by inducing CAV-1-related claudin-5 autophagy.

## Conclusions

Our study suggests that CAV-1-mediated endothelial dysfunction plays a critical role in the pathogenesis of HACE and provides a theoretical basis for the early prevention and treatment of HACE. The findings from this study may offer new insights into the prevention, diagnosis, and timely treatment of HACE. This could reduce the incidence rate, mitigate its damage, and delay the disease process in HACE.

## Supplementary Information

**Additional file 1. Supplementary Figure 1**. CAV-1 regulated the expression of occludin under hypoxic conditions. (A) bEnd.3 cells were exposed to 1% O2 for 24 h. HIF-1α protein levels were detected by Western blot. (B and C) After transfection with siCAV1 for 48 h, bEnd.3 cells were exposed to 1% O2 for 24 h; Occludin protein levels were detected by Western blot (B) and quantified (C) (*p<0.05 and **p<0.01).

## Data Availability

All other data supporting the findings of this study are available from the corresponding authors upon reasonable request.

## Abbreviations

HACE: High-altitude cerebral edema
HH: Hypobaric hypoxia
BBB: Blood‒brain barrier
CAV-1: Caveolin-1
BMECs: Brain microvascular endothelial cells
NRF1: Nuclear respiratory factor 1
AMS: Acute mountain sickness
TEER: Transendothelial electrical resistance
MRI: Magnetic resonance imaging
EB: Evans blue
DMEM: Dulbecco’s modified Eagle’s medium
FBS: Foetal bovine serum
ECGS: Endothelial cell growth supplement
3-MA: 3-Methyladenine
Rapa: Rapamycin
HBSS: Hank’s balanced salt solution
TEM: Transmission electron microscope
