# Supplementary material for: Caveolin-1 accelerates hypoxia-induced endothelial dysfunction in high-altitude cerebral edema
Source: Cell Commun Signal. 2022 Oct 17;20:160. doi: 10.1186/s12964-022-00976-3 (PMC9575296; doi:10.1186/s12964-022-00976-3)

**Supplementary Figure 1.** CAV-1 regulated the expression of occludin under hypoxic conditions. (A) bEnd.3 cells were exposed to 1% O_2_ for 24 h. HIF-1α protein levels were detected by Western blot. (B and C) After transfection with siCAV1 for 48 h, bEnd.3 cells were exposed to 1% O_2_ for 24 h; Occludin protein levels were detected by Western blot (B) and quantified (C) (*p<0.05 and **p<0.01).

**Supplementary Figure 1**


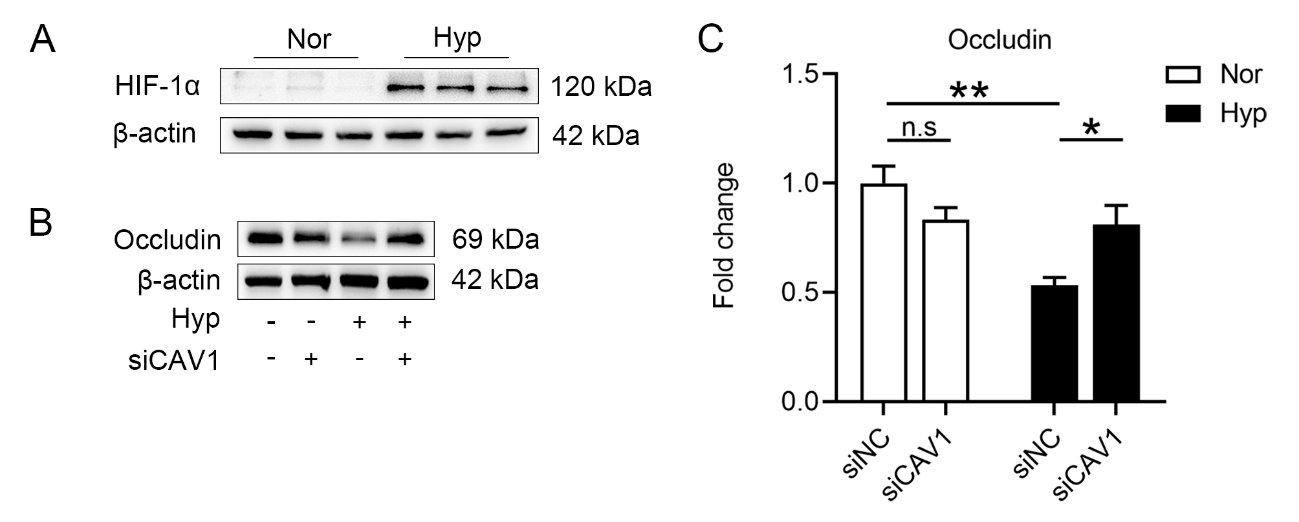

Supplement: Supplementary file 2 — Additional file 1. Supplementary Figure 1. CAV-1 regulated the expression of occludin under hypoxic conditions. (A) bEnd.3 cells were exposed to 1% O2 for 24 h. HIF-1α protein levels were detected by Western blot. (B and C) After transfection with siCAV1 for 48 h, bEnd.3 cells were exposed to 1% O2 for 24 h; Occludin protein levels were detected by Western blot (B) and quantified (C) (*p<0.05 and **p<0.01). [file 12964_2022_976_MOESM2_ESM.docx]
